# High and Low Temperature Performance and Fatigue Properties of Silica Fume/SBS Compound Modified Asphalt

**DOI:** 10.3390/ma13194446

**Published:** 2020-10-07

**Authors:** Xuewen Zheng, Wenyuan Xu, Huimin Feng, Kai Cao

**Affiliations:** 1 School of Civil Engineering, Northeast Forestry University, Harbin 150040, China; 352613703@nefu.edu.cn (X.Z.); fenghuimin_1994@126.com (H.F.); caokai543@163.com (K.C.); 2 China Design Group Co., Ltd., Nanjing 210014, China; 3 College of Civil Engineering and Architecture, Zhejiang University, Hangzhou 310058, China

**Keywords:** silica fume/SBS compound modified asphalt, high temperature performance, low temperature performance, fatigue properties

## Abstract

In order to study the high and low temperature properties, and fatigue properties, of silica fume/SBS (Styrene-Butadiene-Styrene) compound modified asphalt (SFSCMA), dynamic shear rheometer (DSR) and bending beam rheometer (BBR) are used to study matrix asphalt (MA), silica fume modified asphalt (SFMA) (silica fume (SF) 6%), SBS modified asphalt (SBSMA) (mass ratio of SBS to Matrix asphalt 4%), and silica fume/SBS compound modified asphalt, and the high temperature rheological properties of silica fume/SBS compound modified asphalt with different silica fume additions are also studied. The modification mechanism of SFSCMA was studied by scanning electron microscope (SEM). The investigation results turn out: along with the increase in the content of SF, the high temperature performance of SFSCMA is improved significantly. When the content of SF is 6%, the high temperature performance is the best. When the content of SF is more than 6%, the high temperature property of SFSCMA is lower than that of SBSMA. It is suggested to choose 6% as the content of SF. Compared with MA, SFMA, and SBSMA, SFSCMA has excellent high temperature performance; compared with MA and SFMA, the low temperature performance of SFSCMA is improved, but it is worse than that of SBSMA. Moreover, when the temperature is lower than −30 °C, its low temperature performance is close to that of MA, or even worse than that of MA. After the compound modification of SF and SBSMA, the fatigue properties of the asphalt are improved, and the fatigue performance of SFSCMA is the best among the four kinds of asphalt. There is a cross-linking force in the network structure of SFSCMA, which restrains the flow of the whole system, so that the stability of the compound modified asphalt is significantly improved, which is favorable to the high temperature performance and fatigue resistance of the compound modified asphalt. However, due to its low mobility, it has a negative impact on the low temperature performance of the compound modified asphalt. In addition, according to previous studies, compared with diatomite, it is proven that SF can reach the same level as diatomite in improving the high temperature performance and fatigue performance of asphalt. Therefore, SF can be used as a good choice of asphalt modifier and can achieve the purpose of waste recycling and environmental protection.

## 1. Introduction

Silica fume (SF) is an industrial dust produced from ferrosilicon or metal silicon. It has the advantages of chemical corrosion resistance; good thermal stability and dimensional stability, excellent mechanical and electrical properties, and reinforcement. Silica fume (SF) is widely used in building, chemical, metallurgical, and other industries. For example, it can be used as cement or concrete admixture to improve the performance of cement or concrete, and mixing special concrete and compound cement with high strength, wear resistance, erosion resistance, corrosion resistance, permeability resistance, frost resistance and early strength, it has been well used in water conservancy, harbor wharfs, highways, airport runways, tunnels, and other fields. China is rich in silica fume (SF). Because of its unique microporous structure and better adsorption capacity, it has been used as a modifier to make modified asphalt.

SBS (Styrene-Butadiene-Styrene) modified asphalt (SBSMA) is in widespread use, owing to its good road function, but there are many shortcomings in SBSMA [1,2,3,4], for example, SBS is not compatible with matrix asphalt (MA), and it is easy to produce segregation when stored at high temperatures. At the same time, SBS is also a kind of high molecular polymer. Polymers will be degraded under the action of heat, oxygen, light, and mechanical force. Therefore, the degradation of the modifier will accelerate the aging of compound modified asphalt materials to a certain extent, thereby reducing the service life and anti-aging performance of the pavement.

When modified binder is used as a component of the asphalt mixture, the role of “silica fume (SF)” is played by the finest elements of mineral material, based on the excellent properties of SF modification and the shortcomings of SBS modification. In this paper, silica fume (SF) and SBS are added to the MA at the same time for compound modification to explore the performance of silica fume/SBS compound modified asphalt (SFSCMA), in order to promote the development of pavement materials and prolong the service life of pavement.

In past research, the application of silica fume is mainly concentrated in the field of concrete, to improve the mechanical properties of concrete. For example, Erhan Güneyisi [5] et al. used silica fume (SF) instead of cement to prepare concrete containing silica fume. The research results show that the mechanical properties of rubber concrete are improved, and the strength loss rate of concrete is reduced by adding silica fume. Jin Liu [6] et al. prepared a kind of compound mineral admixture on the basis of the mass ratio of steel slag to SF of 84:16. The effect of steel slag- SF compound mineral admixture on the hydration performance of cement-based composite binder, and its concrete performance, were studied. Mansour Fakhri [7] et al. used silica fume instead of eight kinds of cement materials with different contents, to study the influence of waste rubber particles and silica fume on the mechanical properties of roller compacted concrete pavement. The application research of silica fume in concrete is far more extensive than the above examples, and many achievements have been made at home and abroad. However, the research on the application of silica fume in asphalt and its mixture is relatively little, and the research is relatively simple. For example, Yang Song [8] et al. studied SF as an bitumen modifier in the laboratory. It was found that SF can improve the high temperature performance and temperature affection performance of asphalt and improve the high temperature stability and water stability of the mixture. Xu Wenyuan [9] et al. analyzed the modification mechanism of SFSCMA by scanning electron microscopy (SEM) and infrared spectroscopy (FTIR). Luo Zixuan [10] used the viscosity aging index and softening point to evaluate the aging resistance of SFSCMA. Cao Kai [11] et al. studied the influence of SF on the high temperature and low temperature performance of SBS modified bitumen mortar through softening point, viscosity, DSR, and BBR trial. Shang Wenlong [12] evaluated the rheological properties of aged silica fume modified asphalt (SFMA) by DSR test. Rim Larbi [13] et al. used SF as a mineral admixture to improve the compressive strength of rap concrete. Chunfa Ouyang [14] et al. prepared low density polyethylene (LDPE) modified asphalt with high temperature storage stability by adding silica into LDPE. The results show that silica can balance the poor polarity of asphalt and LDPE and improve the high temperature storage stability of asphalt.

In conclusion, there is little research on the fatigue nature and low temperature performance of SFSCMA at home and abroad. Although the research on high and low performance of SFSCMA is involved, the elevated temperature and microtherm property and mechanism are not combined. Therefore, in this paper, based on the existing research, DSR dynamic shear test, BBR bending beam creep test, and LAS (linear amplitude scanning) test are used to study the high and low temperature performance and fatigue performance of SFSCMA.

## 2. Materials and Methods

### 2.1. Materials

The matrix asphalt (MA) used in the test is Panjin 90# bitumen, Liaoning Province, China. The silica fume (SF) is provided by Anshan Anmei International Trade Industrial Development Co., Ltd., Liaoning Province, China. Styrene butadiene styrene block copolymer (SBS) is 4601 SBS modifier produced by China Guangdong Huizhou Li Guangdong Rubber Co., Ltd. In the SFSCMA, the SBS mass fraction is 4%, and the SF of 2%, 4%, 6%, and 8%, respectively, is compounded. The technical performance indexes of SF, MA, and SBSMA are shown in Table 1, Table 2, and Table 3, respectively.

### 2.2. Methods

In the experiment, JRJ-300-I (Shanghai Specimen Model Factory, Shanghai, China) type high speed shear agitation emulsifier was used to prepare SFSCMA. The MA was heated to the melting state, then it was sheared with SBS and dried SF at 170–190 °C at high speed, with a rotating speed of 5000 r/min and a shearing time of 60 min. After shearing, it was stirred evenly for 30 min to prepare SFSCMA. The penetration test, ductility test, softening point test, DSR test, and BBR test were carried out for MA, SBSMA, SFMA, and SFSCMA.

For the penetration and ductility test (JTG E20-2011), three samples of composite modified asphalt with silica fume proportion of 2%, 4%, 6%, and 8% were prepared, respectively. The penetration test temperature was 25 °C, the load was 100 g, and the time was 5 s. The ductility test temperature was 5 °C, the tensile rate was 5 cm/min. For the softening point test (JTG E20-2011), two samples were prepared for each proportion of asphalt samples.

Regarding the Dynamic Shear Rheological (DSR) Test—in this article, a stress control pattern was employed. The stress level was held at 0.1 kPa, and the angular frequency was 10 rad/s. The diameters of the specimen were 25 mm (the thickness is 1 mm) and 8 mm (the thickness is 2 mm) (AASHTO T315-09); two parallel tests were carried out for each proportion of asphalt. 

Regarding the Bending Beam Rheometer (BBR) Test—the creep stiffness modulus S and creep rate m can be acquired through BBR trail, and afterwards the microtherm properties of bitumen can be analyzed by these two parameters. The size of the test piece is length × width × thickness (102 × 12.7 × 6.35 mm) (AASHTO T313-09); two parallel tests were carried out for each proportion of asphalt.

Regarding the LAS (linear amplitude scanning) test—according to AASHTO TP101-14, the LAS test is divided into two parts: frequency scanning and amplitude scanning. In frequency scanning, the frequency scanning range of the sample is 0.2–30 Hz, and the strain level is 0.1%, so as to determine the rheological properties of asphalt and obtain the damage analysis parameters; in the amplitude scanning, the loading frequency is 10 Hz, the loading time is 300 s. The loading amplitude increases linearly from 0.1% to 30.0%, and the test temperature is 25 °C. The specific calculation method is as follows: 

The storage modulus $G^{\prime }\left(\omega \right)$ and the angular velocity ω obtained from the frequency scanning test taken as logarithm, and the formula is obtained as follows:

(1) $$\mathrm{lg}G^{\prime }\left(\omega \right)=m\mathrm{lg}\omega +b,$$

Rheological parameters of asphalt α:

(2) α=1/m,

Damage parameter *D*:

(3) $$D\left(t\right)≅\sum_{i=1}^{N}{\left[\pi r_{t}^{2}\left(C_{i-1}-C_{i}\right)\right]}^{\frac{\alpha }{1+\alpha }}{\left(t_{i}-t_{i-1}\right)}^{\frac{1}{1+\alpha }},$$

where $C_{\left(t\right)}=\frac{\left|G^{*}\right|\left(t\right)}{{\left|G^{*}\right|}_{\mathrm{initial}}}$, $\left|G^{*}\right|\left(t\right)$ is complex shear modulus with time in amplitude scanning, ${\left|G^{*}\right|}_{\mathrm{initial}}$ is initial complex shear modulus, $\gamma_{t}$ is strain at time *t*, and *t* is time.

When time is fixed, *C*_(*t*)_ and *D*_(*t*)_ meet the formula:

(4) $$C_{\left(t\right)}=C_{0}-C_{1}{\left(D\left(t\right)\right)}^{c_{2}}\;(C_{0}=1),$$

Failure $D_{f}$:

(5) $$D_{f}={\left(\frac{C_{0}-C_{peakstress}}{C_{1}}\right)}^{\frac{1}{C_{2}}},$$

where $C_{peakstress}$ is *C*_(*t*)_ corresponding to the maximum stress.

Fatigue equation parameters *A* and *B*:

(6) $$A=\frac{f{\left(D_{f}\right)}^{k}}{k{\left(\pi C_{1}C_{2}\right)}^{\alpha }},$$

where *f* = 10 Hz, *k* = 1 + (1 − *C*_2_) α, *B* = 2α.

Finally, the fatigue equation is obtained:

(7) $$N_{f}=A{\left(\gamma_{max}\right)}^{-B},$$

where $\gamma_{max}$ is the maximum strain.

Take the logarithm of both sides of the fatigue Equation (7) to get:

(8) $$\mathrm{lg}N_{f}=\mathrm{lg}A-B\mathrm{lg}\left(\gamma_{max}\right)$$

Regarding the SEM (scanning electron microscope, Hitachi TM3030, Tokyo, Japan) test—in this experiment, the test temperature was 20 °C, and the relative humidity was 50%. According to the operation and test procedures of TM3030 and JB/T 6842-1993, the SEM test of the initial specimen and different bitumen were carried out.

## 3. Results and Discussion

### 3.1. Results of Basic Technical Indexes of SFSCMA with Different SF Content

Three indicator tests were carried out on the SFSCMA with SF content of 2%, 4%, 6%, and 8%. The test results are shown in Table 4.

It can be seen from Table 4 that, after adding different amounts of SF into SBSMA, along with the increase in SF content, the penetration at 25 °C of SFSCMA decreases, the softening point increases, and the ductility at 5 °C decreases. When the SF content is 6%, the ductility of compound modified asphalt changes little. The test results show that the addition of silica fume can improve the high temperature performance of SBSMA but has a negative impact on the low temperature performance. This conclusion can also be confirmed by G.H. Shafabakhsh et al.’s [15] study. Their research shows that the addition of micro-silicon reduces the penetration, ductility and temperature sensitivity of asphalt, and enhances the softening point of asphalt.

### 3.2. DSR Test Results of SFSCMA with Different SF Addition

**Figure 1:**
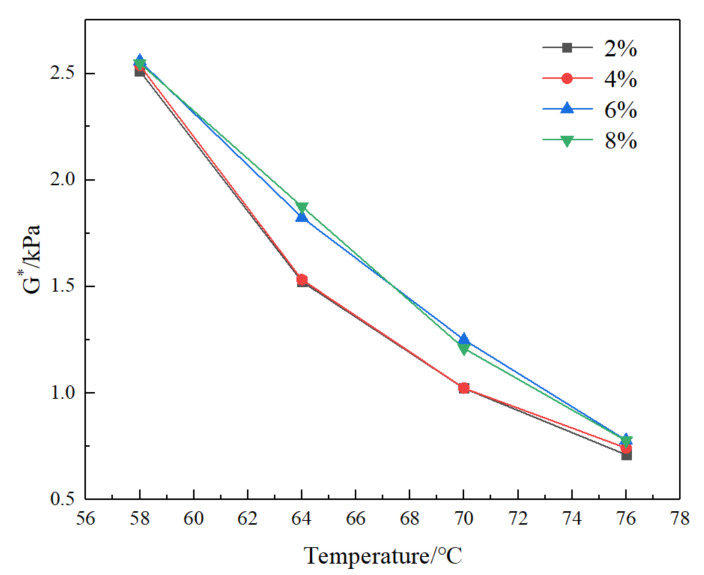
Curves of complex shear modulus G^*^ of bitumen with different SF (silica fume) content with temperature.

**Figure 2:**
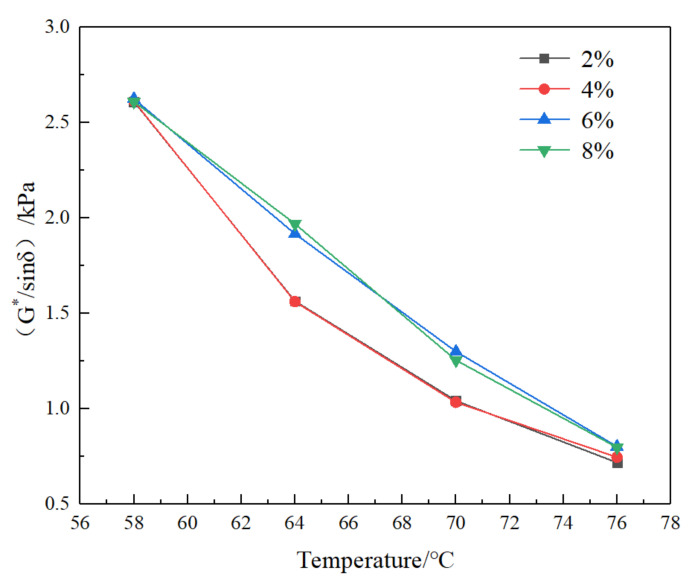
Curves of factor G^*^/sinδ of bitumen with different SF content with temperature.

DSR dynamic shear test of SFSCMA made of SF with 2%, 4%, 6%, and 8% was carried out. The test results are shown in Figure 1 and Figure 2.

Complex shear modulus G^*^ and factor (G^*^/sinδ) are important indexes to describe the high temperature performance of asphalt. The larger the value is, the better the high temperature performance of asphalt is. It can be seen from Figure 1 and Figure 2 that both decrease with the increase in temperature, and increase with the increase in SF content; the curves of SF content at 2% and 4% almost coincide, and the curves of 6% and 8% are relatively close. The results show that (1) with the increase in SF content, the high temperature performance of the compound modified asphalt is improved; (2) when the SF content is 2% and 4%, the high temperature property of the compound modified bitumen is not different, and the high temperature property is close at 6% and 8%. At the same time, in the temperature range of 66–68 °C, the curve of 6% and 8% SF content appears at the intersection, when the temperature is lower than the intersection temperature, the complex shear modulus G^*^ and factor (G^*^/sinδ) of 8% SF compound modified bitumen are slightly higher than those of 6% compound modified asphalt; the complex shear modulus G^*^ and factor (G^*^/sinδ) of 8% SF compound modified asphalt are slightly smaller than those of 6% compound modified asphalt when the temperature is higher than the intersection temperature. This is due to the small bulk density of SF. In the process of preparing asphalt with shear apparatus, SF can be evenly dispersed in asphalt, forming a uniform and continuous network structure with good compatibility, however, when the content of SF is more than 6%, the agglomeration of SF occurs in the process of compatibility with asphalt, which is not conducive to the dispersion of SF, so it affects the compatibility of SF and bitumen, and then affects the high temperature performance of compound modified asphalt. This is basically consistent with [11,16,17] on the content of silica fume. At the same time, research [10] also proposed that when the content of SF is more than 10%, the softening point will decrease, and it is recommended that the content of SF should not exceed 10%. In addition, Hussein H Zghair et al. [18] also verifies that the micro-silica content of 6% has a good effect on improving the physical properties of asphalt. Therefore, combined with the previous research and the research in this paper, the silica fume (SF) content of 6% is recommended.

### 3.3. SHRP PG (Performance Grade) Grade

In order to better evaluate the performance of SFSCMA, MA, SFMA, and SBSMA are selected as the control group. According to the results of 3.1 (three indicators test) and 3.2 (DSR test), 6% SF is selected to prepare silica fume modified asphalt.

According to the SHRP PG grade standard, when the asphalt sample is tested at high temperature, the shear rate is 10 rad/s, which must meet the requirements that the (G^*^/sinδ) value of the original asphalt is not less than 1.0 kPa, the (G^*^/sinδ) value of the residual asphalt after short-term aging (RTFOT) is not less than 2.2 kPa, and the (G^*^/sinδ) value of the residual asphalt after PAV aging is not less than 5000 kPa; when the asphalt sample is tested at low temperature, it must meet the requirements that the creep stiffness S value is not more than 300 MPa, and the creep rate m value is not less than 0.3. The results of SHRP PG grade of four asphalts are given in Table 5, Table 6, Table 7 and Table 8.

It can be seen from Table 5, Table 6, Table 7 and Table 8 that MA, SFMA, SBSMA, and SFSCMA meet the requirements of PG58-16, PG64-16, PG64-22, and PG70-22, respectively.

### 3.4. High Temperature Performance Analysis of Four Kinds of Asphalt

#### 3.4.1. SHRP PG Grade

It can be seen from Table 5, Table 6, Table 7 and Table 8 that, compared with the MA, the high temperature PG grade of the other three kinds of asphalt are improved; the high temperature PG grade of SFMA and SBSMA increases from PG58 to PG64, the high temperature PG grade of SFSCMA increases from PG58 to PG70, and the factor (G^*^/sinδ) of SFSCMA is the largest among the four kinds of asphalts at the same temperature, indicating that it has the best high temperature property.

#### 3.4.2. Complex Shear Modulus G^*^ and Factor (G^*^/sinδ) of Four Kinds of Bitumen

**Figure 3:**
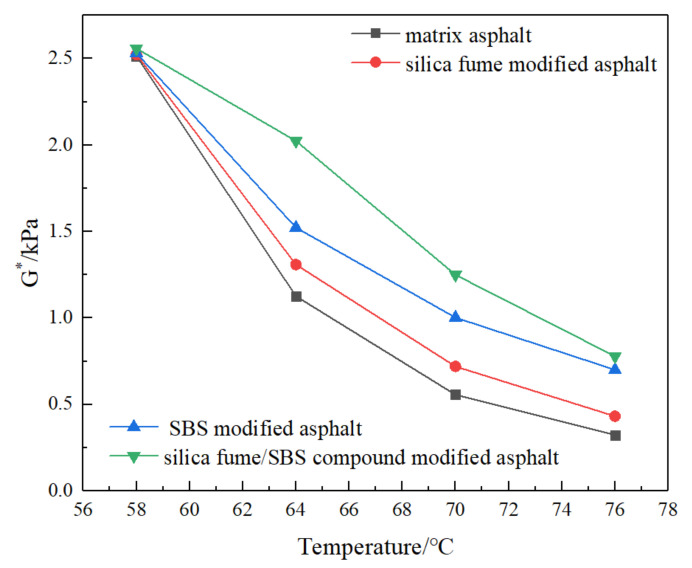
Curves of complex shear modulus G^*^ of four kinds of bitumen with temperature.

**Figure 4:**
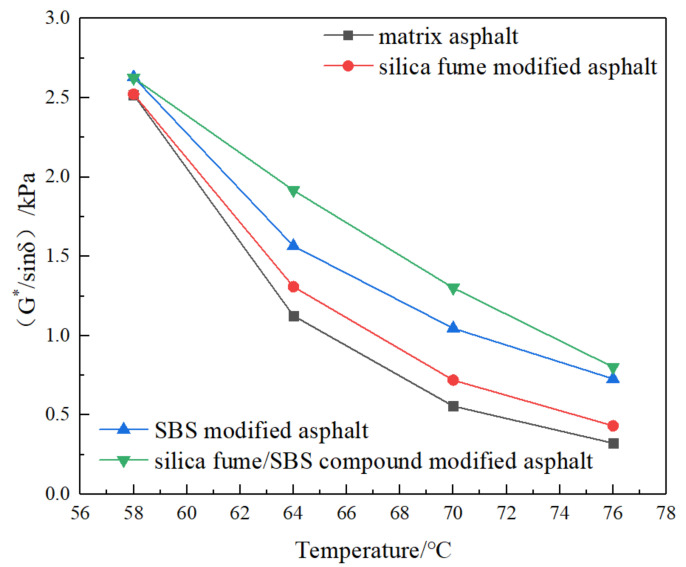
Curves of factor G*/sinδ of four kinds of bitumen with temperature.

The DSR test of MA, SFMA, SBSMA, and SFSCMA is carried out. The test results are shown in Figure 3 and Figure 4.

According to Figure 3 and Figure 4, the complex shear modulus G^*^ and factor (G^*^/sinδ) decreased as the temperature went up. At the same temperature, the complex shear modulus G* and factor (G^*^/sinδ) of SFSCMA are the largest, followed by SBSMA, SFMA, and MA, indicating that SF is beneficial to the elevated temperature property of bitumen, but the elevated temperature performance of SFMA is not as good as SBSMA and SFSCMA. SFSCMA has the best elevated temperature property. This is consistent with the research results of [11,16,19], which show that SF can effectively improve the high temperature performance of asphalt.

#### 3.4.3. Critical Temperature

In Superpave, the ratio of sine value of G^*^ to δ is proposed as the index to evaluate the anti-rut deformation of asphalt material, and it is called the factor (G^*^/sinδ). The bigger the factor is, the stronger the deformation resistance of asphalt is, and the better the high temperature performance is. Through the temperature scanning of DSR, the factors of four kinds of asphalt under different temperatures are obtained. The linear regression analysis is carried out on the curve, and the corresponding temperature when (G^*^/sinδ) = 1 kPa is obtained, which is called the critical temperature. The higher the critical temperature is, the better the high temperature performance of asphalt material is [20]. The test results of the factors are shown in Table 9.

**Figure 5:**
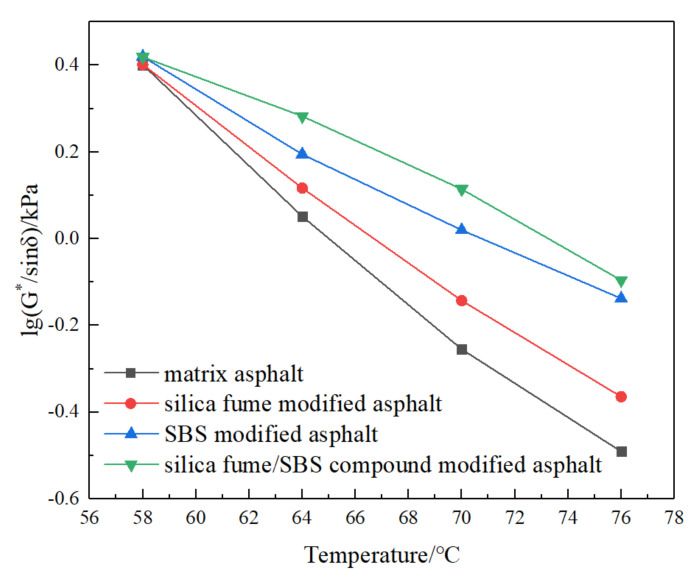
LG (G^*^/sinδ)—T curve of four kinds of asphalt.

According to the factor (G^*^/sinδ) corresponding to different temperature T in Table 9, the (G^*^/sinδ)—T curve is established. For convenience of calculation, take the logarithm of the factor as the dependent variable, and establish the curve of logarithm of factor lg (G^*^/sinδ)—T, as shown in Figure 5.

According to the regression fitting linear equation, we can get the corresponding critical temperature when (G^*^/sinδ) = 1 kPa, that is, lg (G^*^/sinδ) = 0. The results are displayed in Table 10.

It can be seen from Table 10 that the correlation coefficients of four kinds of asphalt are all between 0.980 and 0.996, indicating that the factor lg (G^*^/sinδ) has a good linear correlation with temperature T; After adding SF and SBS modifier, the intercept value of the relation formula is lower than that of the MA relation formula, which shows that the temperature sensitivity of asphalt can be reduced by adding SF. The addition of SF and SBS modifier can improve the critical temperature of the MA. Compared with SBSMA, the critical temperature of SFSCMA is increased by 3.09%, which shows that the high temperature stability of SFSCMA is the best.

#### 3.4.4. High Temperature Performance Evaluation Index Optimization

**Figure 6:**
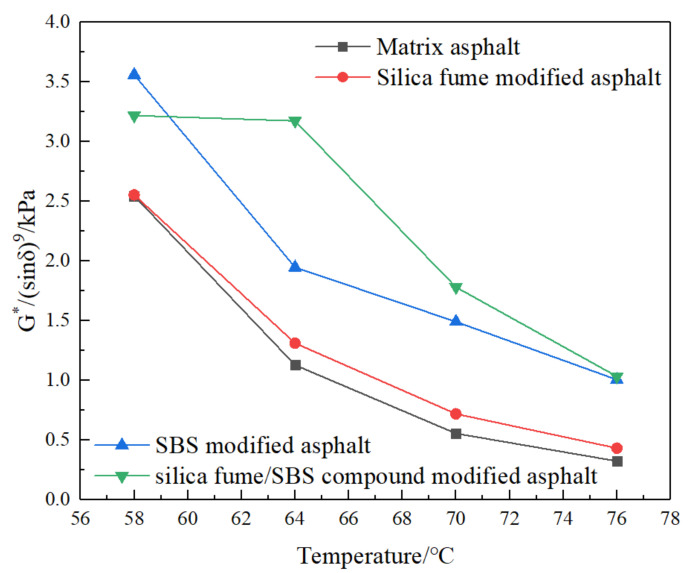
Curves of G^*^/(sin δ)^9^ of four kinds of asphalt with temperature.

It is found that the deformation of asphalt binder is mainly divided into recoverable deformation and nonrecoverable deformation. The factor (G^*^/sinδ) proposed in Superpave has certain limitations in the evaluation of the high temperature performance of asphalt. Therefore, Shenoy [21] proposed to use the nonrecoverable deformation part to evaluate the rut resistance of asphalt. Shenoy and Plazek use the Burgers model to study and find that, in order to make the irrecoverable deformation as small as possible, the phase angle needs to be greater than 58°, so when the phase angle is small, the factor is not applicable. In view of this finding, Shenoy proposes to replace the original factor with the improved factor (G^*^/(sin δ)^9^). The new index (G^*^/(sin δ)^9^) has a phase angle range of 0–90°, which is more sensitive to the change of phase angle, and considers the influence of delayed elasticity, so it can evaluate the viscoelastic behavior of asphalt at different temperatures more comprehensively. Figure 6 shows the curves of improved factor (G^*^/(sin δ)^9^) of four kinds of asphalt with temperature.

It can be seen from Figure 6 that, with the increase in temperature, the improved factors of four kinds of asphalt gradually decrease. Generally speaking, the improved factor of SFSCMA is the largest, followed by SBSMA, SFMA, and MA. It shows that, compared with MA, the addition of silica fume/SBS compound modifier, SBS modifier and silica fume is beneficial to high temperature performance of asphalt, and the high temperature performance of SFSCMA is the best.

### 3.5. Analysis of Low Temperature Performance of Four Kinds of Asphalt

#### 3.5.1. SHRP PG Grade

From Table 5, Table 6, Table 7 and Table 8, it can be seen that both MA and SFMA can meet the requirements at −16 °C, but at the same temperature, the creep stiffness S of SFMA is larger than that of MA, and the creep rate m is smaller than that of MA, so the low temperature performance of SFMA is inferior to that of MA; SBSMA and SFSCMA can meet the requirements at −22 °C, but at the same temperature, the creep stiffness S of SBSMA is smaller than that of SFSCMA, and the creep rate m is larger than that of SFSCMA, so the microtherm property of SBSMA is superior to that of SFSCMA—it shows that adding SF has an unfavorable influence on the microtherm property of bitumen.

#### 3.5.2. Creep Stiffness S and Creep Rate m of Four Kinds of Bitumen

**Figure 7:**
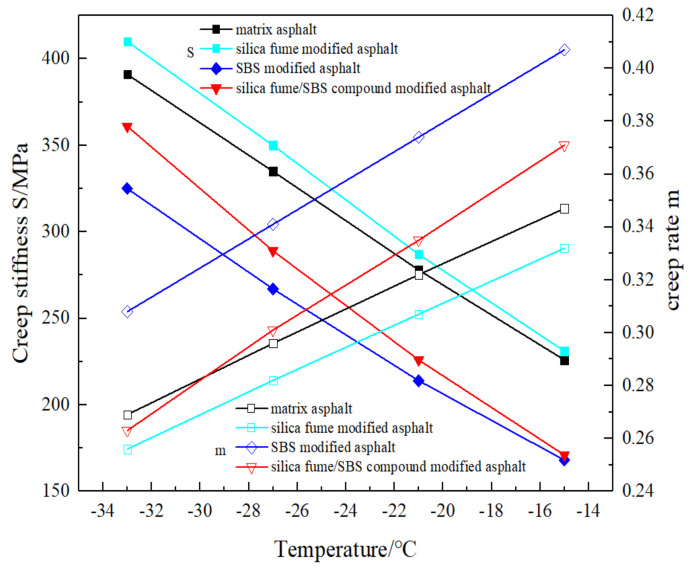
Curves of creep stiffness S and creep rate m of four types of bitumen with temperature.

BBR test was carried out after PAV aging of matrix asphalt, silica fume modified asphalt, SBSMA, and SFSCMA. The test results are displayed in Figure 7.

The creep stiffness modulus S and creep rate m can be acquired by the rheological test of bending beam. These two indexes can well describe the low temperature performance of asphalt. The smaller the S value is, the better the low temperature flexibility is; the larger the m value is, the better the stress relaxation performance and crack resistance performance of asphalt is [22]. It can be seen from Figure 7 that, at the same temperature, the creep stiffness modulus S values of four kinds of asphalt are, in order: SFMA > MA > SFSCMA > SBSMA; when the test temperature is higher than −30 °C, the order of creep rate m is SBSMA > SFSCMA > MA > SFMA; however, when the test temperature is lower than −30 °C, the order of creep rate m is SBSMA > MA > SFSCMA > SFMA. This shows that SBSMA has the strongest relaxation ability and the best low temperature property, followed by SFSCMA, MA, and SFMA; however, when the temperature is higher than −30 °C, the microtherm property of SFSCMA surpasses that of MA. When the temperature is lower than −30 °C, the low temperature property of MA is better than that of SFSCMA. In the temperature range from −33 °C to −15 °C, with the reduction of temperature, the creep stiffness modulus S of four kinds of asphalt increases gradually, while the creep rate m decreases gradually, which indicates that with the decrease in temperature, the microtherm flexibility, the stress relaxation ability and the low temperature crack resistance of four kinds of asphalt decrease.

#### 3.5.3. Low Temperature Crack Resistance Index m/S

**Figure 8:**
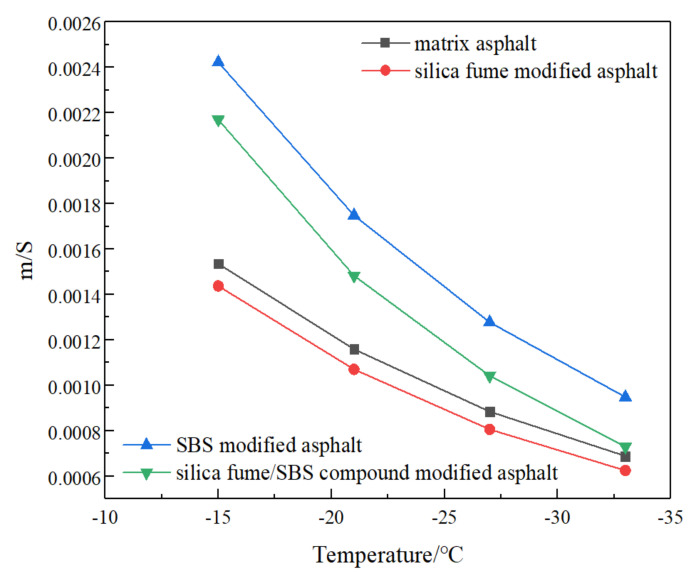
Curves of m/S of four kinds of asphalt with temperature.

To better evaluate the crack resistance of low temperature of four kinds of asphalt, the ratio m/S of creep stiffness S and creep rate m is used for comparative analysis. The larger the m/S is, the better the low temperature crack resistance of asphalt is—otherwise, it is worse. Figure 8 shows the curves of m/S value of four types of asphalt with temperature. It can be seen that in the range of −15 °C to −33 °C, the m/S value of four types of asphalt decreases with the decrease in temperature, indicating that the low temperature crack resistance of four types of bitumen becomes worse with the decrease in temperature. However, at different temperatures, the m/S value of SBSMA is the largest, followed by SFSCMA and MA, and the m/S value of SFMA is the smallest, indicating that the crack resistance at low temperature of SBSMA is the best, the low temperature property of SFSCMA is the second best, and the low temperature performance of SFMA is the worst. In the temperature range of −15 °C to −27 °C, the m/S value of SBSMA and SFSCMA is much higher than that of MA and SFMA; with the decrease in temperature, the m/S values of four types of asphalt are close to each other. When the temperature is lower than −27 °C, the m/S values of SFSCMA and MA are close to each other infinitely. At −33 °C, the values of them are almost the same, indicating that with the decrease in temperature, the difference of their low temperature property is not big. Obviously, compared with the MA, the low temperature property of the SFSCMA is better than that of the MA, but not as good as that of the bitumen mixed with SBS modifier alone. At the same time, research [10] shows that, compared with SBSMA, silica fume has little contribution to the low temperature property of bitumen mixture, and research [16] also shows that the low temperature property of SFMA remains basically unchanged. Research [19] elaborates that low temperature performance of bitumen can be improved when the amount of SF is small, but it has a negative impact on the low temperature with the increase in SF content. Therefore, the research results of microtherm property of SFMA are confirmed in this article.

### 3.6. Analysis of Asphalt Fatigue Performance

#### 3.6.1. Time Scanning

During the DSR test, the attenuation rule of technical indexes of asphalt are obtained by repeated loading, including the complex shear modulus G^*^ and the phase angle δ, so as to determine the fatigue life of asphalt. Some research proposed that the loading times $N_{f50}$ corresponding to the reduction of complex shear modulus to 50% of the initial value is used as the judgment standard of asphalt fatigue failure critical point, which is intuitive and easy to determine, so it is widely used in asphalt fatigue evaluation index systems. According to the research of Sun Daquan [23] et al., it is concluded that $N_{fm}$ > $N_{fG^{*}}$ > $N_{f50}$ > $N_{P20}$ > $N_{1}$ is obtained by using different definitions of asphalt fatigue evaluation indexes, and when the correlation between $N_{fm}$ and $N_{fG^{*}}$ is analyzed, the values of them are close under the same test conditions. Therefore, the number of loading times corresponding to the inflection point of the curve of change rate of complex shear modulus of asphalt in the loading process is taken as a new fatigue life evaluation index, that is, when the complex shear modulus is reduced to 41–44% of the initial value, it is taken as the final fatigue life value. Through DSR time scanning, under the strain control mode, the Sinusoidal oscillation load is applied, and through the analysis of the complex shear modulus, the number of cycles of asphalt to reach the fatigue state is obtained, and the fatigue performance of asphalt is analyzed. Time scanning parameters are shown in Table 11.

The complex shear modulus corresponding to 50 cycles of load cycle is the initial modulus $G_{1}^{*}$, the complex shear modulus corresponding to the fatigue state of asphalt binder is recorded as $G_{2}^{*}$, the times of load when the initial modulus is reduced to 50% are recorded as $N_{f50}$, and the times of load when the initial modulus is reduced to 41–44% are recorded as $N_{fG^{*}}$. The trial results are displayed in Table 12.

As shown in Table 12, under the control strain mode, the complex shear modulus decreases with the increase in the number of cycles, and the asphalt gradually becomes viscous material. Compared with SBSMA, the complex shear modulus and the number of cycles of SFSCMA increases. During the “time scanning” test, the specimen experiences the process of “fatigue damage accumulation–micro crack generation–fatigue damage”, and the addition of silica fume make the fatigue damage accumulation of SBSMA slow down. Therefore, under the same conditions, the fatigue failure time will be later than other asphalts, that is, the more cyclic loading times required, the greater the fatigue life is.

#### 3.6.2. LAS (Linear Amplitude Scanning)

Based on the damage theory of viscoelastic continuum, the LAS (linear amplitude scanning) test consists of frequency scanning and amplitude scanning. In the first stage, the frequency sweep (0.2–30 Hz) was conducted by keeping the applied strain value (0.1%) within LVE range to get the information regarding undamaged material property (α). Thereafter, in the second stage, the binder sample was subjected to amplitude sweep (0–30%) at a frequency level of 10 Hz to capture the binder’s damage property [24]. The specific parameters of test are shown in Table 13.

According to the fatigue model [25,26,27], the fatigue performance of four kinds of asphalt can be evaluated. Table 14 presents the parameters from LAS test for different asphalts. According to the data obtained from the LAS test, the fatigue equation of four kinds of asphalts is obtained through the above model analysis, as shown in Table 15.

Intercept lgA [28] represents the fatigue life of asphalt material, and the larger the value is, the longer the fatigue life is. It can be seen from Table 15 that the intercept of the four kinds of bitumen fatigue equation is in order of SFSCMA > SBSMA > SFMA > MA, and the intercept of SFSCMA fatigue equation is the largest, indicating that it has the longest fatigue life and the best antifatigue performance. Fatigue parameter B is load sensitivity [28], which represents the load sensitivity of material fatigue performance, that is, the performance stability of material under multiple loads. The increase in fatigue parameter B decreases the load sensitivity of the material, so the material has better durability under complex load conditions. Compared with the MA, the B value increases with the addition of SF and SBS. When the asphalt is modified by silica fume and SBS, the B value is the highest, which indicates that the fatigue resistance of the asphalt modified by silica fume/SBS is the best. It shows that the mixing of silica fume can prolong the fatigue life of bitumen and improve the fatigue performance of asphalt.

### 3.7. Modification Mechanism of SFSCMA

**Figure 9:**
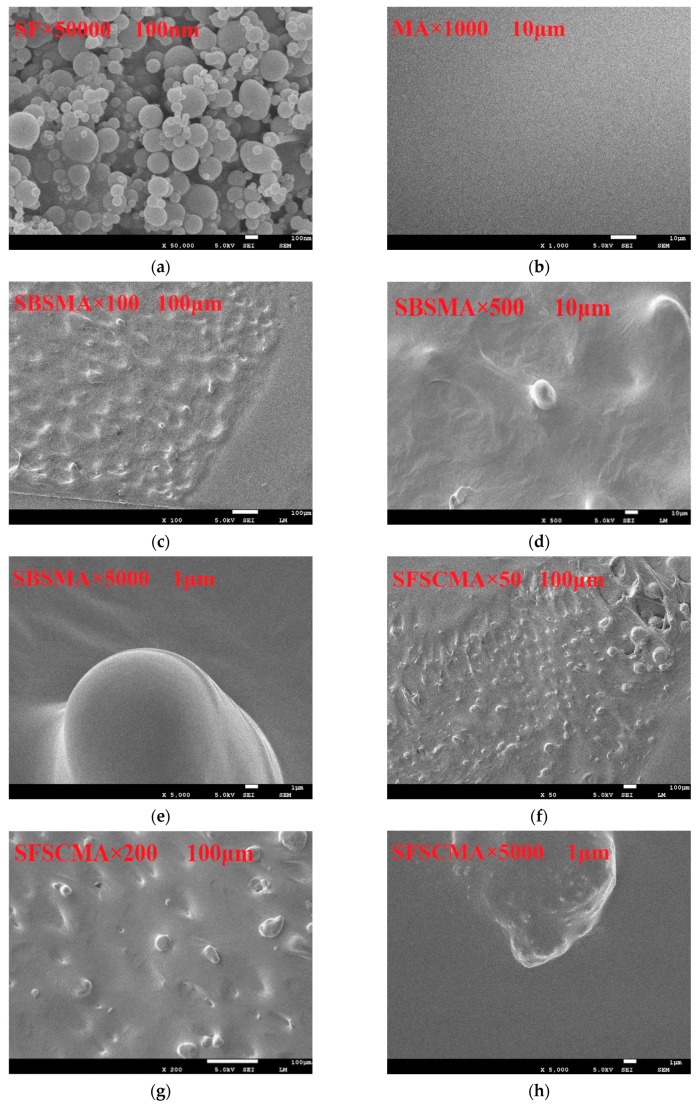
(**a**) SF 50000×; (**b**) MA 1000×; (**c**) SBSMA 100×; (**d**) SBSMA 500×; (**e**) SBSMA 5000×; (**f**) SFSCMA 50×; (**g**) SFSCMA 200×; (**h**) SFSCMA 5000×.

The MA, SFMA, SBSMA, and SFSCMA are scanned by SEM to observe the distribution of silica fume and SBS modifier in the matrix asphalt and explore the modification mechanism of SFSCMA. The test results are displayed in Figure 9a–h.

The premise that the modifier can give full play to its characteristics in the matrix asphalt is that it can be evenly dispersed in the matrix asphalt, so that the whole modified asphalt system can obtain some characteristics of the modifier, so that the modified asphalt can obtain better performance. The distribution of silica fume and SBS in the base asphalt, and the micromorphology of the modified asphalt, can be observed by SEM. The results are displayed in Figure 9a–h. Figure 9a shows that the micromorphology of silica fume is an amorphous sphere with large microgaps between particles and small particle size, about 0.1–1 μm. It has the characteristics of a large specific surface area and strong adsorption. Therefore, it can be evenly dispersed in asphalt in the form of small particles after high-speed shear mixing, forming a certain spatial network structure with matrix asphalt, so as to improve the stability of modified bitumen system. Further, the specific surface area of silica fume is relatively large, which is helpful to enhance the force between silica fume particles and asphalt molecules, make the thickness of structural asphalt film thicker, enhance the adhesion between bitumen and mineral aggregate, and improve the high temperature performance of compound modified bitumen.

It can be seen from Figure 9c that the SBSMA surface is relatively uniform, and SBS is very evenly distributed and adsorbed in the MA. As can be seen from Figure 9d,e, SBS is cut into particles with a particle size of about 10–50 μm under high-speed shear stirring. The particles are evenly dispersed in the MA, forming a stable spatial network structure, which greatly enhances the adhesion between SBS particles and matrix asphalt.

It can be found out from Figure 9f,g that the silica fume particles, SBS particles, and matrix asphalt are well fused into a uniform and stable system, and the silica fume particles and SBS particles are uniformly adhered by the matrix asphalt without agglomeration, which indicates that the compatibility of the three is good. The larger particle size observed in Figure 9f,g,h is about 15 μm, while the particle size of SF is about 0.1–1 μm, and the particle size of SBS is about 10–50 μm as shown in Figure 9a,d; therefore, it can be inferred that the larger particles in Figure 9f,g,h are SBS particles, while the smaller particles are silica fume particles. The interface between SBS particles and matrix asphalt, and the interface between SBS particles and silica fume particles, is very fuzzy, which achieves a good mixing effect.

There is a cross-linking force in the network structure of SFSCMA; the appearance of the network structure indicates the further improvement of the modification effect. The mixing of SF can effectively promote the oil and other light components in the asphalt into the SBS particles, and enhance its swelling degree, which makes the binding ability of SBS particles and asphalt interface further improve. The improvement of binding capacity promotes the formation of cross-linked network structure of SBS in the matrix. The results show that there is no obvious chemical reaction between the modifier and the bitumen in the forced mixing process when silica fume is used for modification, mainly the dispersion, mixing, adsorption and crosslinking between the phases, which is consistent with the research results of [17,29,30]. The cross-linking force between the network and the network will restrict the movement of matrix particles, which greatly reduces the fluidity of the system, and also reduces the segregation of modified asphalt to a certain extent, so that the stability of the compound modified bitumen is significantly improved, and it is favorable to the high temperature property and fatigue resistance of the compound modified asphalt, but it has a negative impact on the low temperature property of the compound modified asphalt due to its low fluidity.

### 3.8. Comparison of Modification of Asphalt with SF and Diatomite

It is well known that SF and diatomite can be used to modify asphalt. The main components of them are silicon dioxide. SF is formed by collecting and processing the smoke and dust escaping from the waste gas in the process of smelting industrial silicon and ferrosilicon in an industrial electric furnace at high temperature. Its particle size is very small, and the average particle size is almost nanometer level. Diatomite is a kind of siliceous rock, which is a kind of biogenic siliceous sedimentary rock. Previous studies have shown that, with the increase in diatomite content, the high temperature performance of bitumen and its mixture can be significantly improved [15,31,32,33,34,35]; however, the research conclusions on the influence of diatomite on the low temperature performance of bitumen are not consistent. Research [31,33,36] shows that the addition of diatomite has a negative impact on the low temperature performance of bitumen, or the impact is very small. However, the study of [32,37,38,39] shows that the diatomite can effectively improve the low temperature performance of bitumen. Therefore, the research on the influence of diatomite on the low temperature performance of bitumen needs further research. Besides, Yu Qian et al. [40] investigated the fatigue performance of diatomite modified asphalt mixture, and the results showed that diatomite can improve the fatigue property of asphalt mixture. This is consistent with the results of this paper. Further, according to [31,32,37], the recommended dosage of diatomite is 10–12%.

Based on the previous research and the experimental study in this paper, it can be seen that for the SF and diatomite whose main components are silicon dioxide, the research of SF in bitumen modification field is far less than that of diatomite, but it can reach the same level as diatomite in improving the high temperature performance and fatigue performance of bitumen, which indicates that SF can be used as a kind of high temperature performance and fatigue performance of modified bitumen. Moreover, SF belongs to industrial waste, which can realize the reuse of resources and achieve the purpose of environmental protection. Moreover, SF has advantages different from diatomite. SF can be directly used for bitumen modification, but the natural diatomite has complex components and needs to be purified before asphalt modification. In addition, the improvement of low temperature performance of asphalt by SF and diatomite is still controversial. It is necessary to further analyze the influence of both on the low temperature performance of bitumen, and to improve the low temperature performance of bitumen by composite modification through relevant means. The adhesion of asphalt is not involved in this paper, and the water stability of asphalt needs to be further studied in the future.

## 4. Conclusions

The following conclusions can be drawn based upon the results obtained:

- The results show that, along with the raise of the content of SF, the high temperature property of SFSCMA is improved. However, when the content of silica fume is more than 6%, the high temperature property of SFSCMA is inferior than that of SBSMA; therefore, combined with the previous research and the research in this paper, the silica fume (SF) content of 6% is recommended.
- The PG grades of MA, SFMA, SBSMA, and SFSCMA are PG58-16, PG64-16, PG64-22, and PG70-22, respectively, which shows that SFSCMA can enhance the PG grade of bitumen and satisfy the use demands of more adverse conditions.
- Compared with MA, SFMA, and SBSMA, the complex shear modulus G^*^, factor G^*^/sinδ, critical temperature and high temperature property index of SFSCMA are the highest, which indicates that the high temperature property of SFSCMA is the best.
- The low temperature property of SFSCMA is superior than that of MA and SFMA, but worse than that of SBSMA, and when the temperature is lower than −30 °C, the microtherm property of SFSCMA is close to or even inferior than that of MA.
- The fatigue performance of SFSCMA is better than that of MA, SFMA, and SBSMA, which shows that the mixing of silica fume can prolong the fatigue life and enhance the fatigue performance.
- In this paper, there is a cross-linking force in the network structure of SFSCMA, which restrains the flow of the whole system, so that the stability of the compound modified asphalt is significantly improved, which is favorable to the high temperature property and fatigue performance of the compound modified bitumen. However, due to its low mobility, it has a negative impact on the low temperature property of the compound modified bitumen.
- Compared with diatomite, it is proven that SF can reach the same level as diatomite in improving the high temperature performance and fatigue performance of asphalt. Therefore, SF can be used as a good choice of asphalt modifier and can achieve the purpose of waste recycling and environmental protection.

## Figures and Tables

**Table 1:** Main technical indicators of SF (silica fume).

Indexes	Results
Appearance	Gray
SiO_2_ content/%	92.88
Particle size distribution/μm	0.1–0.3
Moisture content/%	1.6
Loss on ignition/%	2.8
Specific surface area/(m^2^/g)	25.37
Fineness/%	3
Activity index 28d/%	98.6
PH	6–8

**Table 2:** Main technical indicators of 90# matrix asphalt.

Indexes	Unit	Results	Standard Deviation
25 °C Penetration	0.1 mm	83.6	0.497
Softening Point	°C	48.5	0.200
15 °C Ductility	cm	>100	-

**Table 3:** Main technical indicators of SBS (Styrene-Butadiene-Styrene) modified asphalt.

Indexes	Unit	Results	Standard Deviation
25 °C Penetration	0.1 mm	70.3	0.432
Softening point	°C	61	0.300
5 °C Ductility	cm	32.0	0.616

**Table 4:** The technological indexes of modified asphalt with different mixing amount.

SF Content/%	25 °C Penetration/0.1 mm		Softening Point °C		5 °C Ductility/cm	
	Average Value	Standard Deviation	Average Value	Standard Deviation	Average Value	Standard Deviation
2	79.6	0.779	65.5	0.300	27.9	0.294
4	76.9	0.294	69	0.200	26.7	0.648
6	64.0	0.356	71	0.300	24.8	0.510
8	60.8	0.294	74.5	0.200	24.3	0.572

**Table 5:** PG grade results of MA (matrix asphalt).

Asphalt	Indexes	Temperature/°C	Results	Standard Deviation
Raw asphalt	$\frac{G^{*}}{\sin\delta }$/kPa	58	2.519	0.68%
		64	1.126	0.10%
		70	0.556	0.08%
RTFOT residue	$\frac{G^{*}}{\sin\delta }$/kPa	58	4.711	0.30%
		64	2.160	0.46%
		70	1.030	0.39%
PAV residue	G^*^sinδ/kPa	16	5026	8.40%
		19	4369	10.2%
	Creep stiffness S/MPa	−15	226	5.56%
		−21	278	5.49%
		−27	335	6.10%
	Creep rate m	−15	0.347	0.26%
		−21	0.322	0.15%
		−27	0.296	0.05%
Assessment result	PG58-16			

**Table 6:** PG grade results of SFMA (silica fume modified asphalt).

Asphalt	Indexes	Temperature/°C	Results	Standard Deviation
Raw asphalt	$\frac{G^{*}}{\sin\delta }$/kPa	58	2.523	0.05%
		64	1.309	0.07%
		70	0.720	0.77%
RTFOT residue	$\frac{G^{*}}{\sin\delta }$/kPa	64	2.214	0.07%
		70	1.228	0.26%
		76	0.624	0.38%
PAV residue	G^*^sinδ/kPa	19	5175	7.23%
		22	4231	7.65%
	Creep stiffness S/MPa	−15	231	7.16%
		−21	287	6.31%
		−27	350	5.37%
	Creep rate m	−15	0.332	0.06%
		−21	0.307	0.08%
		−27	0.282	0.06%
Assessment result	PG64-16			

**Table 7:** PG grade results of SBSMA (SBS modified asphalt).

Asphalt	Indexes	Temperature/°C	Results	Standard Deviation
Raw asphalt	$\frac{G^{*}}{\sin\delta }$/kPa	64	1.565	0.04%
		70	1.047	0.72%
		76	0.728	0.07%
RTFOT residue	$\frac{G^{*}}{\sin\delta }$/kPa	64	2.960	0.07%
		70	1.600	0.09%
PAV residue	G^*^sinδ/kPa	22	5225	8.43%
		25	4445	8.47%
	Creep stiffness S/MPa	−21	214	5.95%
		−27	267	5.23%
		−33	325	6.05%
	Creep rate m	−21	0.374	0.05%
		−27	0.341	0.05%
		−33	0.308	0.07%
Assessment result	PG64-22			

**Table 8:** PG grade results of SFSCMA (silica fume/SBS compound modified asphalt).

Asphalt	Indexes	Temperature/°C	Results	Standard Deviation
Raw asphalt	$\frac{G^{*}}{\sin\delta }$/kPa	64	2.625	0.14%
		70	1.917	0.11%
		76	1.300	0.08%
RTFOT residue	$\frac{G^{*}}{\sin\delta }$/kPa	70	2.191	1.07%
		76	1.145	0.10%
PAV residue	G^*^sinδ/kPa	22	5442	5.68%
		25	4080	5.01%
	Creep stiffness S/MPa	−21	226	4.82%
		−27	289	5.02%
		−33	361	4.69%
	Creep rate m	−21	0.335	0.06%
		−27	0.300	0.14%
		−33	0.263	0.03%
Assessment result	PG70-22			

**Table 9:** Factors (G^*^/sinδ) of four kinds of asphalt.

Temperature/°C	(G^*^/sinδ)/kPa			
	MA	SFMA	SBSMA	SFSCMA
58	2.519	2.523	2.630	2.625
64	1.126	1.309	1.565	1.917
70	0.556	0.720	1.047	1.301
76	0.323	0.432	0.728	0.802

**Table 10:** Critical temperature of four kinds of asphalt.

Asphalt	Relation	R^2^	Standard Deviations	Critical Temperature/°C
MA	y=−0.04971x+3.25726	0.98903	0.00164	65.53
SFMA	y=−0.04265x+2.86037	0.99541	0.00050	67.07
SBSMA	y=−0.0308x+2.18778	0.98952	0.00060	71.03
SFSCMA	y=−0.02855x+2.09313	0.98624	0.00068	73.31

**Table 11:** Time scanning test parameters.

Test Parameters	Loading Mode	Strain Control	Temperature/°C	Loading Frequency
parameters	Strain control mode	10%	25	10

**Table 12:** Asphalt fatigue life test results.

Asphalts	$G_{1}^{*}/\mathrm{kPa}$	$G_{2}^{*}/\mathrm{kPa}$	$N_{f50}/\mathrm{times}$	$N_{fG^{*}}/\mathrm{times}$
MA	3980	1667	2451	-
SFMA	4323	1771	2762	2813
SBSMA	5169	2131	11085	13556
SFSCMA	5553	2336	14006	17428

**Table 13:** LAS test parameters.

Parameters	Frequency Scanning	Amplitude Scanning
Load frequency/Hz	0.2–30	10
Strain level	0.1%	-
Loading time/s	-	300
Temperature/°C	25	25

**Table 14:** Parameters from LAS test for different asphalts.

Asphalt	A	B	C_0_	C_1_	C_2_	α
MA	103.983	2.432	1	0.1769	0.5517	1.216
SFMA	345.327	2.604	1	0.1086	1.0136	1.302
SBSMA	666.657	2.689	1	0.0061	0.991	1.344
SFSCMA	1196.604	2.710	1	0.0112	1.086	1.355

**Table 15:** Asphalt fatigue equation.

Asphalt	Fatigue Equation
MA	$\mathrm{lg}N_{f}=2.017-2.432\mathrm{lg}\left(\gamma_{max}\right)$
SFMA	$\mathrm{lg}N_{f}=2.538-2.604\mathrm{lg}\left(\gamma_{max}\right)$
SBSMA	$\mathrm{lg}N_{f}=2.824-2.689\mathrm{lg}\left(\gamma_{max}\right)$
SFSCMA	$\mathrm{lg}N_{f}=3.078-2.710\mathrm{lg}\left(\gamma_{max}\right)$
